# Pharmacological and Non-Pharmacological Methods of Labour Pain Relief—Establishment of Effectiveness and Comparison

**DOI:** 10.3390/ijerph15122792

**Published:** 2018-12-09

**Authors:** Iwona Czech, Piotr Fuchs, Anna Fuchs, Miłosz Lorek, Dominika Tobolska-Lorek, Agnieszka Drosdzol-Cop, Jerzy Sikora

**Affiliations:** 1 Department of Pregnancy Pathology, Department of Woman’s Health, School of Health Sciences in Katowice, Medical University of Silesia, 40-752 Katowice, Poland; afuchs999@gmail.com (A.F.); cor111@poczta.onet.pl (A.D.-C.); jerzy_sikora@poczta.onet.pl (J.S.); 2 Student’s Scientific Organisation of Gyneacology, Obstetrics and Sexology, Medical University of Silesia, 40-752 Katowice, Poland; fuchspiotr@gmail.com (P.F.); lorek.milosz@gmail.com (M.L.); tobolska.d@gmail.com (D.T.-L.)

**Keywords:** labour pain, water immersion, TENS, epidural anaesthesia, pain relief

## Abstract

*Background*: To evaluate the effectiveness of pharmacological and non-pharmacological pain relief methods and to compare them. *Materials and methods*: 258 women were included in the study and interviewed using a questionnaire and the visual analogue scale for pain. They were divided into six groups depending on chosen method of labour pain relief: epidural anaesthesia (EA; *n* = 42), water immersion and water birth (WB; *n* = 40), nitrous oxide gas for pain control (G; *n* = 40), transcutaneous electrical nerve stimulation (TENS) (*n* = 50), multiple management (MM; *n* = 42), none (N; *n* = 44). *Results*: The average age of the women was 29.4 ± 3.74 years and 60.47% of them were nulliparous (*n* = 156). Mean values of labour pain intensity were 6.81 ± 2.26 during the first stage of labour; 7.86 ± 2.06 during the second stage, and 3.22 ± 2.46 during the third stage. There was no significant difference in pain level between epidural analgesia and gas groups in the first stage of labour (*p* = 0.74). Nevertheless, epidural analgesia reduced pain level during the second and third stage (both *p* < 0.01). The highest satisfaction level pertains to water immersion (*n* = 38; 95%). *Conclusion*: Epidural analgesia is the gold standard of labour pain relief, however water birth was found to be associated with the highest satisfaction level of the parturient women. The contentment of childbirth depends not only on the level of experienced pain, but also on the care provided to the parturient during pregnancy and labour.

## 1. Introduction

The experience of childbirth is a subjective and multidimensional issue and each woman passes through it in a different way. It is one of the most beautiful episodes in a mother’s life, associated with joy, happiness, and celebration. However, a delivery is also related to negative emotions: fear, anxiety, low sense of security, and the expectation of pain [1]. Women often mention, that because of their anxiety they would prefer a caesarean section rather than a natural delivery [2]. Occasionally, women feel little if any pain in labour and give birth unexpectedly [3].

A scientific definition of pain is ‘an unpleasant sensory and emotional experience associated with actual or potential tissue damage’ [4]. Labour pain has its two elements: visceral and somatic. The visceral one occurs during the first stage of labour and it is connected with the tension exerted on the cervix, which causes its dilatation. That is felt by a parturient as a pain. The somatic kind of pain appears at the end of the first stage and it lasts also in the second stage. It is a result of the force exerted on the vaginal part of the cervix, the vagina and the perineum [5]. Pain stimulates the respiratory system, minute ventilation and oxygen consumption increase and hyperventilation may cause respiratory alkalosis and reduction in the amount of blood transported to the foetus [6]. Moreover, pain, anxiety, and stress during delivery can cause an increased release of catecholamines and cortisol into the circulation [7]. However, we cannot forget, that pain during delivery plays an essential role in informing about labour’s progress. Among factors that influence the severity of pain, we can recognize the individual’s pain threshold, the level of labour’s progress, size of the baby, the mother’s general health condition, pelvic dimensions, position during the delivery, stress and mental factors [8].

Labour pain management is not only a crucial concern for future mothers but also a great challenge in modern medicine. A wide range of both pharmacological and nonpharmacological labour pain relief techniques are currently available for pregnant women in Poland. The first group includes: epidural analgesia, gas for pain control and intravenous opioids. Nonpharmacological techniques contain water birth and water immersion, transcutaneous electrical nerve stimulation (TENS), aromatherapy, acupuncture and acupressure, massage techniques [9]. Currently a neuraxial blockade including a spinal, epidural, or a combined spinal-epidural technique is considered to be the gold standard for labouring patients [2]. However, many of the parturient patients also appreciate non-pharmacological methods of pain relief, because it can allow them to experience the delivery in a more natural way.

It is important to educate future mothers about the process of natural delivery and possible pain relief methods in order to increase their satisfaction connected with the delivery [1,2,5]. Most of the parturients expect to obtain effective pain relief during the delivery [10] and the goal in obstetrics practice is to choose a method that will reduce the pain to a level in which parturients are able to cope with it, and simultaneously to give the parturient the possibility to participate in the birth experience.

The aim of this work is to establish women’s experience of labour pain, to compare pharmacological and non-pharmacological pain relief methods and to evaluate the effectiveness of each chosen technique. This work tries to find a gold solution in labour pain relief.

## 2. Materials and Methods

### 2.1. Study Group

The study sample was drawn from the Center of Woman’s and Child’s Health in Zabrze, Silesia, Poland. The eligibility criteria included: gestational age >37 weeks, single foetus, cephalic position of the foetus, spontaneous onset of labour, appropriate uterine contractions, physiological pregnancy, and age >18 years. Exclusion criteria were: hypersensitivity for any of the products used, or contraindications for epidural analgesia. There were 608 labours in the Center in the period from September 2016 to February 2017. Out of them, 371 women delivered a baby naturally. After the selection process we enrolled into the study 258 women who met the inclusion criteria, aged from 18 to 39 years (29.34 ± 3.74).

All included women were divided into six groups, depending on the chosen method of labour pain relief: epidural anaesthesia (EA; *n* = 42), water immersion and water birth (WB; *n* = 40), gas for pain control (G; *n* = 40), TENS (*n* = 50), multiple management (MM; *n* = 42), none (N; *n* = 44). In our study multiple management was defined as a combination of gas with water immersion or gas with TENS method.

The ethics committee of the Medical University of Silesia, Katowice, Poland abolished the requirement of having their permission for conducting the study (KNW/0022/KB/280/17).

### 2.2. Questionnaire

The author’s questionnaire contained 21 questions and it was divided into two parts: The first one referred to the demographics, past medical history and infant birth outcomes, while the second one consisted of questions concerning chosen pain management techniques and assessment of pain level during each stage of labour with the use of visual analogue scale for pain (VAS). Each woman was interviewed personally, up to 48 h after the delivery. The questionnaire was firstly pretested on the group of 20 women, who were asked to comment on the clarity of the questions.

#### 2.2.1. Demographics and Infant Birth Outcomes

A number of demographic data were examined during the study including age, the number of pregnancies and labours, social status, education level, attendance at the neonatal classes, sex of the neonate. Infant birth outcomes such as birth-weight, birth-length, head circumflex and Apgar score were also checked in the medical history. A requirement for episiotomy was noted in each case. Women were also asked if they had performed the perineum massage in the antenatal period.

#### 2.2.2. Past Medical History

Women were asked to give information about the diseases diagnosed before and during pregnancy. Additionally, women provided details about medications taken during the pregnancy.

#### 2.2.3. Methods of Labour Pain Management and Pain Assessment

Women were asked to indicate chosen technique of labour pain relief. The list of methods included pharmacological treatment (epidural analgesia, nitrous oxide gas for pain relief), non-pharmacological pain relief therapy (TENS, water immersion/water birth) and no pain relief. Women were able to choose more than one technique (ex. gas and TENS). In case of epidural analgesia women were additionally asked to provide information of any side effects of this method.

Due to selection bias, one should not ignore the roles of pain and analgesia on subsequent childbirth satisfaction. Women with longer, more difficult, more complicated labours are more likely to have analgesia or to choose various pain management techniques.

Experienced pain during each stage of labour was assessed on a visual analogue scale for pain (VAS) [11], where severity is marked on a scale with a range from 1 to 10, where 0 refers to no pain and 10 illustrates the worst possible, unbearable, excruciating pain. Women were also asked about how much labour pain they had expected before the delivery.

A satisfaction from chosen technique was measured by a question “Would you repeat this method in your next labour?”

#### 2.2.4. Statistical Analysis

All data analyses were conducted using StatSoft Statistica version 13.0 PL software and *p* value < 0.05 was considered as significant. Shapiro-Wilk test was utilized to verify the normality of variables. The results were analysed using the following statistical tests: one-way ANOVA, Kruskall-Wallis ANOVA, Mann-Whitney *U* test and regression analysis. For qualitative variables, a chi^2^ test was used.

## 3. Results

The average age of the women was 29.4 ± 3.74 years and 60.47% of them were nulliparous (*n* = 156). Overall anthropometrical and clinical characteristics of Study and Control groups women were comparable (Table 1).

**Figure 1:**
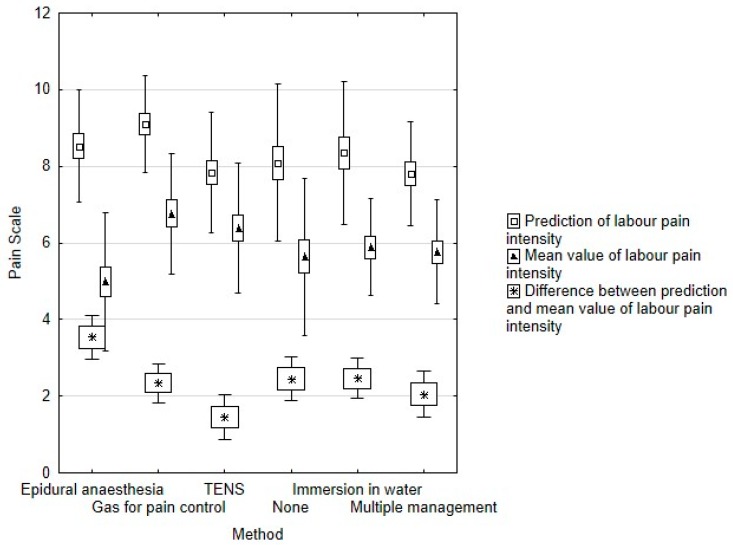
Association between prediction and mean value of labour pain intensity.

Women expected the forthcoming childbirth to be painful, with the mean prediction of labour pain intensity (PLPI) being 8.27 ± 1.65 and these results did not statistically differ among particular groups (*p* = 0.054, Figure 1).

The impact of parity to pain assessment is presented in Table 2. Multiparas expected higher pain intensity (*p* < 0.01) and experienced stronger pain in the second and third stage of labour (*p* = 0.03 and *p* = 0.01 respectively).

We have identified significant positive correlation between PLPI and the following factors: age (r = 0.26; *p* < 0.01), gestational age (r = 0.20; *p* = 0.04) and child’s weight (r = 0.17; *p* = 0.03).

The majority of parturients had higher education (*n* = 172; 66.67%), there were 62 women with secondary education (24.03%) and equally 12 women (4.65%) had primary education and vocational qualification. The education status did not affect the mean value of labour pain intensity (MLPI; *p* = 0.26), however women with primary education had statistically lower PLPI (5.16 ± 2.48; *p* = 0.01) and most of them did not choose any pain relief method (*n* = 6; 50% in this group). On the other hand, women with higher education tended to choose TENS or combined analgesia (both *n* = 38; 22.09%; *p* = 0.028).

Our analysis has revealed that there were no differences in sense of pain at any stage of labour between women that attended parental craft classes and women who did not (*p* > 0.05). Perineum massage did not contribute to reduction of a frequency of perineal incision (*p* > 0.05). Episiotomy also did not affect pain intensity of our study subjects (*p* > 0.05). Among all women who had episiotomy (*n* = 112; 43.41%), the vast majority were nulliparous (*n* = 88; 78.57%) (*p* < 0.01).

The mean values of labour pain intensity were 6.81 ± 2.26 during the first stage of labour (LPI I); 7.86 ± 2.06 during the second stage (LPI II); and 3.22 ± 2.46 during the third stage (LPI III). The MM group was the only group in which we have observed a reduction of pain intensity between the second and the first stages of labour (7.23 ± 2.07 and 7.62 ± 2.20 respectively). The results of pain intensity at different stages of labour between particular pain relief methods are presented in Table 3.

Out of the women, 82.81% were satisfied with their chosen pain relief method (*n* = 212) and 79.69% claimed that they would repeat the chosen method in the future (*n* = 204). Regardless of the chosen method, women who had an episiotomy were more dissatisfied with their pain management technique (*p* = 0.01). There was also a negative correlation between mean value of pain intensity and satisfaction level (r = −0.37; *p* < 0.01).

### 3.1. Pharmacological Methods

**Figure 2:**
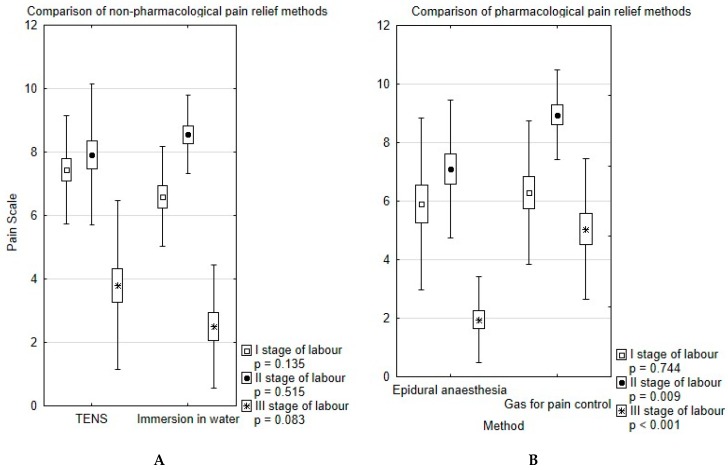
Comparison of non-pharmacological (**A**) and pharmacological (**B**) pain relief methods.

Figure 2 displays the comparison between pharmacological and non-pharmacological methods.

There was no significant difference in pain level between EA and G groups in the first stage of labour (*p* = 0.74). Nevertheless, EA reduced pain level during the second and third stage (both *p* < 0.01). Additionally, the biggest difference between PLPI and MLPI was reported in this group (3.54 ± 1.84; *p* = 0.02; Figure 1). We have observed two cases of iron deficiency anaemia as a complication of EA.

### 3.2. Non-Pharmacological Methods

We did not observe statistical differences in the pain level experienced between TENS and WB groups at any stage of labour (Figure 1). The highest satisfaction level pertains to water immersion (*n* = 38; 95%), while only 68% of parturients would choose TENS in their next labour (*n* = 34) (*p* = 0.024).

## 4. Discussion

### 4.1. Epidural Analgesia

Studies clearly show that epidural analgesia is still the most effective and most often recommended method of pain relief during labour [6,12,13]. Our study remains consistent with this state, as pain severity was the lowest in each stage of labour among women who chose this method. In addition, in the second and third stages of labour it was a statistically better technique than nitrous oxide. However, we should underline that this may be connected with the fact that gas for pain relief was associated with the highest levels of pain in these stages of labour. Furthermore, in the group of patients who chose EA, the difference between the level of expected pain and the mean value of experienced pain was the biggest in comparison with other groups. Literature does not remain consentaneous in the case of frequency of instrumental vaginal delivery. Some studies show, that in the case of EA during childbirth there is a greater need of performing instrumental vaginal delivery [14], while others demonstrate that the instrumental delivery rate for the whole obstetric population may not be changed by the introduction of an epidural analgesia use [15]. The risk of chronic back pain and caesarean section is not increased, although instrumental vaginal delivery may be more likely [16].

Although the incidence of complications from epidural analgesia during labour seems to be low, women may still feel concerned about the side effects of EA. Among all interviewed women in our research (*n* = 42), two cases of deficiency anaemia as a complication of EA were identified. Even though a headache and low back pain are commonly reported complications after epidural anaesthesia, none of our patients experienced it.

### 4.2. Gas for Pain Control

Nitrous oxide (N_2_O) gas is a commonly used labour analgesic in many Western countries [17]. N_2_O is a nonflammable, tasteless, odourless gas. It was firstly used as a labour analgesic by Stanislav Klikovich in Poland in 1881. Klikovich published the results of his study wherein he utilized 80% N_2_O with 20% oxygen in 25 labouring women, and demonstrated pain relief with no adverse foetal outcomes [18]. When comparing inhalation analgesia with placebo or no treatment, nitrous oxide was found to offer more pain relief [6]. However, inhalation of nitrous oxide was found to provide less effective pain relief than epidural analgesia [19]. Some studies show that the majority of pregnant women receiving the nitrous oxide were satisfied with its analgesic effects and reported good experiences about it [20]. In our study, the use of nitrous oxide alone referred to the lowest reduction in severity of pain among all methods. In addition, it was a statistically worse pain-reliever in the second and third stage of labour than the epidural analgesia. However, a combination of gas with TENS or water immersion (multiple management) demonstrated better results and this issue will be discussed in a further part of this study.

### 4.3. Water Immersion and Water Birth

In recent days, a method that has grown in popularity because of its numerous advantages is water birth. It is a technique that has been used since the ancient times and in Poland the first water birth took place in 1996 in Łódź and the first labour with water immersion was in Tychy [21,22]. Various physiological effects of this mode of delivery help explain its beneficial impact. Women who used this method have reported feeling safe, relaxed and in control [23]. Because of the hydrostatic pressure, the intraabdominal pressure is higher and that eases the future mother in breathing and changing position [24]. In addition, water enables the pelvic tissues to be more flexible and elastic and that reduces the pain during the contractions and the number of instrumental interventions during the delivery [25]. In the present study, water birth and/or water immersion were not related to statistical reduction of pain severity. The group of the parturients who chose this method established their experience of pain as lower in stage I and II, and higher in stage II in comparison to women who chose the other non-pharmacological method, TENS. However, the results were not statistically important. A fact that should be underlined is that in our study, although water birth did not statistically reduce the level of pain, it was a well-accepted technique and it was associated with the highest satisfaction among the parturients. That may be associated with relaxing influence of water [26]. Satisfaction is a multidimensional and complex issue and it is hard to establish it. Studies show, that factors like personal expectations, support from caregivers, the relationship between the parturient and the caregiver and the possibility of involvement in the process of making decisions play the most crucial role [27,28]. Some other studies also mention effective analgesia as an important factor [28] and the outcomes of our study are compatible with this theory, as patients who felt less severe pain were more satisfied from labour than those who suffered from more harmful pain. Recently, an increasing popularity in involving the patients in the decision-making process has been observed, also in obstetrics practice [29]. Doctors should be encouraged to take into consideration women’s suggestions, as this may be a fundamental factor in their experience. Every parturient has expectations, and identifying women’s expectations, demands, choices, and anxieties help the health care providers to reach their goal, which in this case is a positive birth experience [30]. An individual, supportive approach to each patient should be provided in order to ameliorate women’s experience of labour.

### 4.4. Transcutaneous Electrical Nerve Stimulation (TENS)

Transcutaneous electrical nerve stimulation is a popular method of pain relief that has been used in Europe for 30 years. Although some studies show that it is an alternative and useful method for labour analgesia [31], our study is not consistent with this statement. Neither did it reduce labour pain effectively, nor did it meet with recognition of the parturients because TENS was the method that was identified as the least satisfying. 32% (*n* = 34) of women who chose this technique in our study were not willing to repeat it in the future. However, when TENS was combined with gas for pain control, a better reduction in severity of pain was observed. Moreover, in our study TENS was associated with the smallest difference between the predicted intensity of pain and the mean value of labour pain. However, when TENS was combined with gas for pain control, a better reduction in severity of pain was observed. Some authors claim that TENS reduces pain especially during the first stage of labour, and when used later, it may minimize or postpone the necessity of pharmacological analgesia [31], while others do not agree with the last statement [32].

### 4.5. Multiple Management

Multiple management was the only approach that reduced the pain in comparison with the previous stages. Further studies should be performed in order to investigate the effectiveness of using one main method of pain relief consolidated with an additional one. Alternative methods such as aromatherapy, massage techniques, acupuncture, music therapy, and yoga should be remembered, as these could be important adjuncts to the major analgesic method [6,33]. Some studies suggest, that complementary medicine may be useful for the early onset of pain or as a distracter by redirecting the parturient’s attention from the source of pain. These studies also underline the fact that the degree of success of a used technique is correlated with the availability of support staff in both educational and trial phases of the studies, and necessarily in clinical practice. Although the medical staff does not need to be specialized in the topic of alternative labour pain relief therapies, they should have a basic knowledge in this subject and they should be able to give women advice concerning further information because the need for alternative therapies in obstetrics will be rising [34]. Nowadays, acupuncture tends to be more widely used to manage labour pain. As a complementary medical modality, it has been reported to be effective in improvement of certain painful conditions such as labour pain. Recent studies support the efficacy of acupuncture and acupressure in decreasing labour pain and duration, although debate continues [7,35]. Using non-invasive techniques, obstetricians can achieve not only pain relief but also an extremely important factor—reduction of anxiety during labour which helps to encourage the mothers to have a vaginal delivery.

There were no statistically significant differences in felt pain between women who underwent the episiotomy and those who did not. The massage of the peritoneal area during pregnancy did not have an impact on the frequency of episiotomy. However, some studies show that antenatal perineal massage reduces the occurrence of perineal trauma (mainly episiotomies) and the reporting of ongoing perineal pain and is generally well accepted by women [36]. Despite the results of our study in this area, we still have to encourage women to perform the massage of the perineal area and teach them how to do it properly. According to the recommendations of the Polish Society of Gynecologists and Obstetricians, it is a method of prevention from injuries of the perineum and their consequences [37]. In addition, the Polish Society of Gynecologists and Obstetricians indicates that primiparas are more likely to have episiotomy or injury of the peritoneum [37] and that statement is consistent with the results obtained in our work. A fact that has to be underlined is that in our study women who had an episiotomy were less satisfied with their labour. That once more indicates the necessity of good perinatal healthcare.

Surprisingly, we found out that in the second and in the third stage of labour, multiparas experienced stronger pain than primiparas and the results were statistically important. Probably this result could be explained by the analysis of the chosen pain relief methods among these both groups, but unfortunately, we were not able to investigate the parity effect respectively to each pain relief method due to insufficient quantity of patients in the groups. A study performed on a larger group of women could give us more answers. Some studies show that parity is not associated with the use of epidural analgesia [10]. Another explanation of this result could be women’s earlier experience, as previous deliveries could have an impact on multiparas’ perception of pain during current childbirth.

In our study, there were no statistically important differences in the established mean value of pain between women who took part in the antenatal classes and those who did not. This kind of preparation for delivery has become widespread, especially in developed countries. Studies present that the main need of the expecting parents is to enlarge their knowledge about the process of delivery, parenthood and to make them feel more secure in taking care of their infant [38]. Studies show, that antenatal classes have positive effects on both mother’s and child’s health and that women who are well-informed and self-conscious about their pregnancy are less likely to undergo obstetric surgery, they need fewer medical procedures and they are more satisfied with their experience of labour. The only condition is that they have to be able to apply the knowledge attained at the antenatal classes during the childbirth [39].

## 5. Conclusions

Our study remains consistent with the statement that epidural anaesthesia remains the gold standard of pain relief. Nitrous oxide, when used alone, refers to lowest pain reduction among all methods, but when it is used in a combination with TENS or water immersion it diminished the severity of pain in following stages of labour. However, we should remember that besides experienced level of pain, it is also the attention and care provided during the antenatal and neonatal period, which determines the level of satisfaction from labour. Among all chosen techniques in our study, water immersion remains the most acceptable non-pharmacological pain relief method, contrary to TENS which was associated with the lowest level of satisfaction. In addition, it is important to educate parturients about available pain relief methods in labour and to help them to cope with their fears associated with childbirth.

## Figures and Tables

**Table 1:** Clinical characteristics of delivering women stratified by chosen pain relief method.

	Epidural Anaesthesia (*n* = 42)	Gas for Pain Control (*n* = 40)	TENS (*n* = 50)	Immersion in Water (*n* = 40)	Multiple Management (*n* = 42)	*p* Value
Age (years)	29.95 ± 3.69	28.00 ± 3.51	29.20 ± 3.83	29.40 ± 3.91	30.00 ± 3.24	0.07
Number of Pregnancies (*n*)	1 (1–4)	2 (1–3)	1 (1–2)	1 (1–3)	1 (1–3)	0.53
Number of Labour (*n*)	1 (1–2)	1.5 (1–3)	1 (1–2)	1 (1–3)	1 (1–3)	0.09
Education						0.31
Primary	0	2 (5)	2 (4)	2 (5)	0	
Secondary	14 (33.33)	14 (35)	10 (20)	4 (10)	2 (4.76)	
Vocational	2 (4.76)	2 (5)	0	4 (10)	2 (4.76)	
Higher	26 (61.9)	22 (55)	38 (76)	30 (75)	38 (90.48)	
Living and housing condition						<0.01
Weak	0	0	0	0	0	
Average	4 (9.52)	4 (10)	2 (4)	2 (5)	0	
Good	14 (33.33)	24 (60)	30 (60)	12 (30)	10 (23.8)	
Very good	24 (57.14)	12 (30)	18 (36)	26 (65)	32 (76.19)	
Timing of childbirth (weeks)	39.19 ± 1.12	39.40 ± 0.82	39.20 ± 1.15	39.45 ± 1.05	39.33 ± 0.73	0.87
Child’s weight (g)	3485.24 ± 300.92	3574.50 ± 352.11	3364.6 ± 437.31	3293.5 ± 308.82	3324.76 ± 388.31	0.54
Child’s height (cm)	54.95 ± 2.06	54.50 ± 5.26	54.12 ± 2.80	54.00 ± 2.41	52.38 ± 7.92	0.08
Child’s HC (cm)	33.35 ± 1.09	34.21 ± 1.08	34.04 ± 0.89	33.28 ± 1.04	33.34 ± 1.40	0.85
APGAR—1 min	10 (9-10)	10 (7-10)	10 (9-10)	10 (9-10)	10 (9-10)	0.38
APGAR—5 min	10 (9-10)	10 (8-10)	10 (10-10)	10 (9-10)	10 (10-10)	0.56

Data are presented as mean ± SD, *n* (%) or median (range). Transcutaneous electrical nerve stimulation (TENS), Head circumference (HC).

**Table 2:** Comparison of pain assessment between primaparas and multiparas.

	Primapara (*n* = 156)	Multipara (*n* = 102)	*p* Value
PLPI	8.04 ± 1.44	8.61 ± 1.88	<0.01
MLPI	5.83 ± 1.63	6.02 ± 1.83	0.50
Difference between PLPI and MLPI	2.20 ± 2.06	2.59 ± 1.71	0.14
LPI I	6.86 ± 2.28	6.74 ± 2.24	0.66
LPI II	7.67 ± 2.07	8.16 ± 2.00	0.03
LPI III	2.98 ± 2.45	3.59 ± 2.43	0.01

Data are presented as mean ± SD. Prediction of labour pain intensity (PLPI), mean value of labour pain intensity (MLPI), labour pain intensity during the first stage of labour (LPI I), labour pain intensity during the second stage of labour (LPI II), labour pain intensity during the third stage of labour (LPI III).

**Table 3:** Comparison of pain assessment at different stages of labour stratified by chosen pain relief method.

	Epidural Anaesthesia (*n* = 42)	Gas for Pain Control (*n* = 40)	TENS (*n* = 50)	Immersion in Water (*n* = 40)	Multiple Management (*n* = 42)	None (*n* = 44)	*p* Value
PLPI	8.52 ± 1.45	9.10 ± 1.24	7.84 ± 1.56	8.35 ± 1.85	7.81 ± 1.35	8.09 ± 2.02	0.06
MLPI	4.98 ± 1.79	6.77 ± 1.55	6.39 ± 1.68	5.88 ± 1.25	5.76 ± 1.35	5.64 ± 2.02	0.04
Difference between PLPI and MLPI	3.54 ± 1.84	2.33 ± 1.56	1.45 ± 2.05	2.47 ± 1.65	2.05 ± 1.94	2.45 ± 1.92	0.02
LPI I	5.90 ± 2.91	6.30 ± 2.42	7.44 ± 1.69	6.60 ± 1.55	7.62 ± 2.20	6.86 ± 2.19	0.16
LPI II	7.10 ± 2.34	8.95 ± 1.52	7.92 ± 2.19	8.55 ± 1.22	7.23 ± 2.07	7.47 ±2.11	0.02
LPI III	1.95 ± 1.45	5.05 ± 2.36	3.80 ± 2.63	2.50 ± 1.91	2.43 ± 2.26	3.55 ± 2.59	<0.01

Data are presented as mean ± SD.

## Abbreviations

EA	Epidural analgesia
G	Gas for pain control
HC	Head circumference
LPI I	Mean values of labour pain intensity in the first stage of labour
LPI II	Mean values of labour pain intensity in the second stage of labour
LPI III	Mean values of labour pain intensity in the third stage of labour
MLPI	Mean value of labour pain intensity
MM	Multiple management
N	No pain relief
PLPI	Mean prediction of labour pain intensity
TENS	Transcutaneous electrical nerve stimulation
VAS	Visual analogue scale
WB	Water birth

## References

[1] Karlsdottir S.I., Sveinsdottir H., Olafsdottir O.A., Kristjansdottir H. (2015). Pregnant women’s expectations about pain intensity during childbirth and their attitudes towards pain management: Findings from an Icelandic national study. Sex Reprod. Healthc..

[2] Serçekuş P., Okumuş H. (2009). Fears associated with childbirth among nulliparous women in Turkey. Midwifery.

[3] Gaskin I.M. (2008). Ina May’s Guide to Childbirth: Updated with New Material.

[4] Niven C., Gijsbers K. (1984). A study of labour pain using the MCGILL pain questionnaire. Soc. Sci. Med..

[5] Labor S., Maguire S. (2008). The Pain of Labour. Rev Pain..

[6] Koyyalamudi V., Sidhu G., Cornett E.M., Nguyen V., Labrie-Brown C., Fox C.J., Kaye A.D. (2016). New Labor Pain Treatment Options. Curr. Pain Headache Rep..

[7] Asadi N., Maharlouei N., Khalili A., Darabi Y., Davoodi S., Shahraki H.R., Hadianfard M., Jokar A., Vafaei H., Kasraeian M. (2015). Effects of LI-4 and SP-6 Acupuncture on Labor Pain, Cortisol Level and Duration of Labor. J. Acupunct. Meridian Stud..

[8] Bręborowicz G. (2015). Położnictwo i Ginekologia.

[9] Sulima M. (2013). Alternatywne metody łagodzenia bólu porodowego. Eur. J. Med. Technol..

[10] Lindholm A., Hildingsson I. (2015). Women’s preferences and received pain relief in childbirth—A prospective longitudinal study in a northern region of Sweden. Sex Reprod. Healthc..

[11] Huskisson E. (1974). Measurement of pain. Lancet.

[12] Freeman L.M., Bloemenkamp K.W., Franssen M.T., Papatsonis D.N., Hajenius P.J., van Huizen M.E., Bremer H.A., van den Akker E.S., Woiski M.D., Porath M.M. (2012). Remifentanil patient controlled analgesia versus epidural analgesia in labour A multicentre randomized controlled trial. BMC Pregnancy Childbirth.

[13] Howell C.J. (1999). Epidural versus non-epidural analgesia for pain relief in labour. Cochrane Database Syst. Rev..

[14] Howell C.J., Kidd C., Roberts W., Upton P., Lucking L., Jones P.W., Johanson R.B. (2001). Randomised controlled trial of epidural compared with non-epidural analgesia in labour. Br. J. Obstet. Gynaecol..

[15] Impey L., MacQuillan K., Robson M. (2000). Epidural analgesia need not increase operative delivery rates. Am. J. Obstet. Gynecol..

[16] Kinsella S.M. (2001). Epidural analgesia for labour and instrumental vaginal delivery: An anaesthetic problem with an obstetric solution?. BJOG Int. J. Obstet. Gynaecol..

[17] Collins M.R., Starr S.A., Bishop J.T., Baysinger C.L. (2012). Nitrous oxide for labor analgesia: Expanding analgesic options for women in the United States. Rev. Obstet. Gynecol..

[18] Richards W., Parbrook G.D., Wilson J. (1976). Stanislav Klikovich 1853–1910. Anaesthesia.

[19] Likis F.E., Andrews J.C., Collins M.R., Lewis R.M., Seroogy J.J., Starr S.A., Walden R.R., McPheeters M.L. (2014). Nitrous oxide for the management of labor pain: A systematic review. Anesth. Analg..

[20] Certosimo F., Walton M., Hartzell D., Farris J. (2002). Clinical evaluation of the efficacy of three nitrous oxide scavenging units during dental treatment. Gen. Dent..

[21] Reid-Campion M. (1997). Hydrotherapy: Principles and Practice.

[22] Pawelec M., Pietras J., Wojtysiak M., Karmowski M., Karmowski A., Palczynski B., Terpiłowski Ł. (2011). Water birth and water immersion—An important step towards more patient-oriented health care or a new way for obstetrical wards to make profits?. Adv. Clin. Exp. Med..

[23] Benfield R.N., Hortobágyi T., Tanner C.J., Swanson M., Heitkemper M.M., Newton E.R. (2010). The effects of hydrotherapy on anxiety, pain, neuroendocrine responses, and contraction dynamics during labor. Biol. Res. Nurs..

[24] Poder T.G., Larivière M. (2014). Bénéfices et risques de l’accouchement dans l’eau: Une revue systématique. Gynécol. Obs. Fertil..

[25] Zanetti-Daellenbach R.A., Tschudin S., Zhong X.Y., Holzgreve W., Lapaire O., Hösli I. (2007). Maternal and neonatal infections and obstetrical outcome in water birth. Eur. J. Obstet. Gynecol. Reprod. Biol..

[26] Juda W., Madej M., Zalewski M., Jerzy H., Zalewski J. (2012). Personal experiences of water birth. Zdr Publ..

[27] Hodnett E.D. (2002). Pain and women’s satisfaction with the experience of childbirth: A systematic review. Am. J. Obstet. Gynecol..

[28] Spaich S., Welzel G., Berlit S., Temerinac D., Tuschy B., Sütterlin M., Kehl S. (2013). Mode of delivery and its influence on women’s satisfaction with childbirth. Eur. J. Obstet. Gynecol. Reprod. Biol..

[29] Say R.E., Thomson R. (2003). Clinical review decisions—Challenges for doctors. Br. Med. J..

[30] Goldbort J.G. (2009). Women’s Lived Experience of Their Unexpected Birthing Process. Mcn Am. J. Matern. Nurs..

[31] Santana L.S., Gallo R.B.S., Ferreira C.H.J., Duarte G., Quintana S.M., Marcolin A.C. (2015). Transcutaneous electrical nerve stimulation TENS reduces pain and postpones the need for pharmacological analgesia during labour: A randomised trial. J. Physiother..

[32] Mello Larissa F.D., Nóbrega L.F., Lemos A. (2011). Transcutaneous electrical stimulation for pain relief during labor: A systematic review and meta-analysis. Braz. J. Phys. Therapy.

[33] Smith C.A., Collins C.T., Cyna C.M., Crowther C. (2003). Complementary and alternative therapies for pain management in labour. Cochrane Database Syst. Rev..

[34] Tournaire M., Theau-Yonneau A. (2007). Complementary and alternative approaches to pain relief during labor. Evid.-Based Complement Altern. Med..

[35] Levett K.M., Smith C.A., Dahlen H.G., Bensoussan A. (2014). Acupuncture and acupressure for pain management in labour and birth: A critical narrative review of current systematic review evidence. Complement Ther. Med..

[36] Beckmann M.M., Stock O.M. (2013). Antenatal perineal massage for reducing perineal trauma. Cochrane Database Syst. Rev..

[37] Ginekologicznego R.Z.E.P.T. (2011). Dotyczące Zapobiegania Śródporodowym Urazom Kanału Rodnego oraz Struktur dna Miednicy. Ginekol Pol..

[38] Ahldén I., Ahlehagen S., Dahlgren L., Josefsson A. (2012). Parents’ expectations about participating in antenatal parenthood education classes. J. Perinat. Educ..

[39] Spinelli A., Baglio G., Donati S., Grandolfo M.E., Osborn J. (2003). Do antenatal classes benefit the mother and her baby?. J. Matern. Neonatal. Med..

